# A Great Achievement

**Published:** 1875-11

**Authors:** 


### A GREAT ACHIEVEMENT.

Last month we prepared a supplementary edition of HALL'S JOURNAL OF HEALTH, and printed more than 50,000 copies of it. We did this mainly in order to give wide knowledge of the fact that we were ready to make excellent terms with new subscribers. We called this edition the "American Institute Edition," and intended to distribute the tons of interesting reading matter to visitors of that attractive exhibition, but we saw that those visitors were fairly overburdened with printed show-bills and descriptive circulars, which they received only to destroy; and we determined not to add our contribution to the general holocaust. We decided that we would have our little pamphlet distributed in a way which would secure for it attention and preservation. We preferred that our extra should be left at the best houses in the great city of New York. Of course we knew it to be a great work to quickly call upon 50,000 of the best families in this city, and present each head of the house with our book. Some of our friends told us that the work would last until next Spring, even though followed up on every pleasant day. We wanted the thing done in one day, and we secured its completion *in one hour!* And this is how we did it.

We employed the AMERICAN DISTRICT TELEGRAPH COMPANY to put for a single hour, nearly its whole force upon our distribution. During this hour it barely reserved men and boys enough to attend to imperative calls at the several district offices. All the rest, over five hundred messengers, devoted their energies to our business. The work was done, and faithfully done. The cost to us was $100, or $2 per thousand. We could not have hired it done at all perfectly, for a much larger sum, in any other way. We could not find anybody else who would promise to do the work in anything short of one month. And yet it was swept off and finished up in a single hour, and the pamplets were placed just where we wished them placed.

This Company has divided the city into 27 districts, and in each of these districts it has an office. Here are stationed competent men, and an army of uniformed boys. In our case, the pamphlets were delivered to Mr. Ashhurst, Supt. Messenger Department of the Company, 62 Broadway, from whence they were divided up among the district offices, from Union Square to Harlem. From these district offices they were, at a given moment, taken by the requisite number of messengers, to the residences previously agreed upon. No other establishment in America could have done this work, and done it efficiently, in the same sixty minutes.

The business of delivering letters, circulars, books, packages, etc., is by no means the chief business of the American District Telegraph Co. It has electrical wires running from its several offices to hotels, public edifices, and private residences. Each wire terminates in an instrument by the aid of which the occupant of the place can instantly register a signal in the district office. By touching the key the citizen says to the officers at the district office—"send me a messenger"—and within three minutes the messenger is by his side. By another signal he can say, "send me a Policeman," and the Policeman appears as quickly. By still another preconcerted signal, the work of an instant, the demand is made for the Fire Brigade of the Company, and the force is at hand, with the speed of the wind, and fully provided with the means of putting out a fire. All this is true of each and every hour of the twenty-four. The offices never close; a part of the messengers being engaged by day, and others by night. The system is seemingly perfect, and has become a necessity in all the better houses of New York.

We went out the other evening to test its efficiency. An officer of the Company accompanied us, but our movements were not pre-arranged. We summoned here a messenger, and there a policeman; we imagined a fire at remote points, and signalized the Company's fire department, standing, meantime, watch in hand, to determine whether much of a conflagration could occur before the arrival of means to extinguish it. In every case we were surprised at the almost instantaneousness of the response. We terminated our investigations, abundantly convinced of the efficiency of the service, in all its departments.

Nearly all the messengers are boys, ranging from fourteen to sixteen years. Some have been in the employ of the Company since its organization, three years ago. They are all under rigid military discipline. In encountering a superior in rank, they touch the hat in military style. Their uniform is tasteful, and is kept in perfect order. These boys live at home, but they are not allowed to wear their uniform home, or when off duty. They leave home in their citizens dress, and change in the Company's dressing-rooms. They are taught good manners and good morals. They are not allowed to linger by the way, or to converse with anybody. The office time-table tells them how long they may be absent on an errand, and they must make the trip on time, or give a satisfactory explanation of the detention. They receive $4 per week for the service imposed. They look bright, and happy, and healthy, and seem greatly to enjoy this active life.

The cost attending this system is of course, enormous. It is repaid by the fees of the messengers, which are small in themselves, but large in the aggregate, and by the rental of the instruments of which we have spoken. Each of these instruments rents for $2,50 per month. Their use is extending daily, on account of the admitted efficiency of the service. One of our friends, who has an instrument in his library, tells us that it has proved itself worth hundreds of dollars to him, by enabling him to summon his physician at the dead of night; others feel that their premises have been saved by the use of the instrument to call the means of extinguishing accidental fires.

This is a "modern improvement," and one of the most useful and extensive known to us. It will go on extending its usefulness until we shall wonder how we ever managed to live without it.

The American District Telegraph Company of New York, is the originator and parent of this system which we have spoken of, and has organizations of the same nature in all the chief cities of the country.

---

### PEARLED WHEAT.

Our friends and neighbors, the Health Food Company, have recently added to their list of valuable foods, an article which they call Pearled Wheat. It is simply choice white, winter Wheat, made clean, and polished, so that nearly all its coarse outer hull is removed. In this form it is put up in boxes of two sizes, which sell, respectively for 25 cents and sixty cents each. It is used precisely as cracked wheat, or crushed wheat, or wheaten grits are used, and is certainly the finest thing of the kind we have ever seen. It is very easily cooked, as all that it requires is to be let alone after mixing abundant water with it, and exposing it to sufficient heat to cook but not burn it. Eaten with sugar and cream it is a "dish for the gods,"

and the time was when no higher praise than this could be bestowed. Sliced up cold and eaten in milk, or fried, it far surpasses the cracked wheat dishes in use. Or, by making a batter of the excellent Attrition Flour and mixing the cold pearled-wheat mush with it and then baking or otherwise cooking it, a splendidly palatable and nutritious food is secured.

Now wheat, even raw wheat, if it is clean and sound, is a wonderfully valuable food. It has been the most fearfully abused substance in the world, by injurious grinding and cooking processes; but it is, in its best estate, the noblest general food known. We have kept a box of this pearled wheat on our office-table for two weeks past, and have invited patients and callers to partake of it in its raw state. In this way we have relieved a number of bad cases of constipation and dyspepsia, and headache, caused by weak stomachs. It would be well for this class of sufferers to try this method of cure.

